# Altered cortical synchronization in photosensitive idiopathic generalized epilepsy

**DOI:** 10.1002/epi.70425

**Published:** 2026-08-13

**Authors:** Alessandro Benedetto, Cecilia Steinwurzel, Francesco Turco, Enrica Bonanni, Roberto Cantello, Gionata Strigaro

**Affiliations:** ^1^ Department of Neurosciences, Psychology, Drug Research and Child Health (NEUROFARBA) University of Florence Florence Italy; ^2^ Department of Translational Research on New Technologies in Medicine and Surgery University of Pisa Pisa Italy; ^3^ Epilepsy Center, Neurology Unit University of Piemonte Orientale and Azienda Ospedaliero‐Universitaria “Maggiore Della Carità” Novara Italy

**Keywords:** alpha variability, cortical excitability, phase coherence, photosensitive epilepsy, SSVEP

## Abstract

**Objective:**

To characterize cortical excitability and synchronization dynamics in patients with idiopathic generalized epilepsy (IGE) and photosensitivity by assessing steady‐state visual evoked potentials (SSVEPs) elicited by intermittent photic stimulation.

**Methods:**

SSVEPs were recorded from the occipital cortex (Oz) in patients with IGE and photoparoxysmal response and in age‐matched healthy subjects (HSs) during stimulation at 8 and 16 Hz, with eyes open and closed. We analyzed evoked (phase‐locked) and induced (non–phase‐locked) responses, inter‐trial phase clustering (ITPC), broadband electroencephalographic energy, and resting‐state spectral and alpha‐peak dynamics.

**Results:**

Compared with HSs, patients showed consistently greater electroencephalographic energy at stimulation frequencies and across the broadband spectrum, particularly for induced SSVEP responses. Despite this increased amplitude, patients exhibited reduced phase coherence, reflected by significantly lower ITPC across all conditions. Resting‐state analyses revealed heightened broadband power but unstable alpha oscillations, with greater variability in individual alpha‐peak frequency. Together these findings indicate enhanced cortical responsiveness accompanied by impaired temporal precision in neural synchronization.

**Significance:**

Photosensitive IGE is characterized not only by exaggerated visual cortical excitability but also by disrupted synchronization dynamics, suggesting a state of strong but temporally unstable neural activity that may facilitate transitions toward photically induced paroxysms. SSVEP‐based measures and alpha‐variability metrics may serve as functional biomarkers of network instability in photosensitive epilepsy.

**Key points**

- Patients with photosensitive idiopathic generalized epilepsy (IGE) showed increased broadband electroencephalographic responses during rhythmic visual stimulation.
- Visual responses were stronger in patients but showed reduced inter‐trial phase coherence (ITPC).
- Reduced ITPC suggests impaired neural temporal precision despite enhanced cortical responsiveness.
- Resting electroencephalography showed increased broadband power and greater alpha‐peak frequency variability.
- Steady‐state visual evoked potentials and alpha‐variability measures may provide biomarkers of cortical network instability.

## 1 INTRODUCTION

Cortical excitability, defined as the cortex's responsiveness to external stimuli, is a fundamental determinant of neuronal networks and a critical parameter in epilepsy research. Changes in cortical excitability are associated with alterations in neuronal synchrony, which typically reflect shifts between physiological and pathological network states.^1^ Epileptogenicity is characterized by episodes of hypersynchronous neuronal firing that manifest as paroxysmal events, such as seizures or interictal epileptiform discharges (IEDs).^2^ However, recent evidence indicates that these manifestations are more complex, with hypersynchrony representing a late stage of a more nuanced pathological network activity. A phase of cortical desynchronization often precedes hypersynchrony and can predict the occurrence of focal seizures.^3^, ^4^ This desynchronization, possibly reflecting compensatory inhibitory mechanisms,^5^ suggests that epileptic dynamics may result from an abnormal network response to (internal or external) perturbations, associated with atypical fluctuations in cortical excitability.^6^ Although hypersynchrony can be identified by visual inspection of spontaneous electroencephalography (EEG) as high‐amplitude sharp activity, altered temporal coordination, and band‐specific power require frequency and phase analysis of perturbed EEG signals, that is not routinely performed in clinic.

Stimulation‐based approaches, which evoke measurable responses to sensory input, provide a unique window into these cortical processes by allowing controlled probing and modulation of cortical networks, and may also reveal altered excitability even when no obvious epileptic discharges are visible on EEG.^7^ Among sensory modalities, visual stimulation is particularly relevant for eliciting the photoparoxysmal response (PPR), a well‐established example of stimulation‐induced paroxysmal activity.^8^ PPRs can be reliably triggered by trains of intermittent photic stimulation (IPS) during routine EEG recordings, and most commonly observed in idiopathic generalized epilepsies (IGEs), particularly juvenile myoclonic epilepsy (JME).^9^ Notably, rhythmic stimulation is a key factor in the genesis of PPRs, with flashes flickering at ~15 Hz representing the most effective stimulus for eliciting PPRs in photosensitive IGE in clinical practice.^8^

Several factors contribute to the generation of the PPR in photosensitive IGE, including cortical hyperexcitability in response to one or two photic stimuli,^10^ and an abnormal functional visuo‐motor network, that mediates rapid propagation of paroxysmal activity from posterior to anterior motor areas.^11^ This network dysfunction can facilitate epileptic motor phenomena, such as myoclonus.^10^, ^11^, ^12^, ^13^ Visual evoked potentials (VEPs) to transient stimuli are also altered in PPR^14^; however, single‐pulse stimuli rarely induce seizures, making them useful but limited probes of the epileptogenic circuit. Several studies report that transient VEP amplitudes (e.g., P100) do not consistently differ between photosensitive patients and controls, suggesting that transient and steady‐state measures may capture complementary aspects of cortical function rather than redundant information.^15^, ^16^

Although one‐ or two‐pulse photic stimulation can help in characterizing early cortical responses,^10^ it is particularly informative to examine how the PPR develops during trains of photic stimulation resembling IPS during routine EEG recordings. To this end, steady‐state visual evoked potentials (SSVEPs)—the steady cortical responses to fast periodic visual stimulation—provide access to the dynamics of neural populations under continuous excitatory drive, a condition that more closely resembles the initial stages of a seizure trigger in photosensitive epilepsy, and have been used to demonstrate impaired inhibitory control in the occipital cortex of these patients.^14^ Nevertheless, despite their clear advantages, SSVEPs remain largely unexploited in epilepsy research. Recent studies have begun to explore SSVEPs in epilepsy,^16^, ^17^ providing important initial insights into visual cortical responses. However, these investigations were based on standard clinical EEG protocols, in which photic stimulation is delivered as single continuous trains of several seconds per frequency, resulting in a limited number of trials and a higher likelihood of inducing PPRs or seizures. In contrast, we propose an approach based on multiple brief and repeated stimulation trains at each frequency, designed to enhance the detection of the hyperexcitability endophenotype. This trial‐based design allows a more refined characterization of temporal response patterns and cortical excitability, while reducing the risk of triggering paroxysmal activity.

In this study, we used SSVEPs to investigate visual cortical excitability in patients with IGE and photosensitivity (IGE + P), compared to age‐matched healthy subjects (HSs). We recorded EEG activity both at rest and during IPS at 8 and 16 Hz, with eyes open/closed.

We hypothesized that patients would exhibit hyperexcitability, even in the absence of hypersynchronous paroxysmal discharges, reflected in altered SSVEPs responses and spontaneous brain activity. By examining evoked, induced, and spontaneous brain dynamics, we aim to characterize a neurophysiological phenotype of photosensitive epilepsy that extends beyond the mere occurrence of PPR, potentially establishing SSVEPs as a functional biomarker of system excitability dysfunction in these patients.

## 2 MATERIALS AND METHODS

Data pre‐processing and analyses were performed with the MATLAB toolbox EEGLAB, FieldTrip,^18^ and custom‐made code.

### 2.1 Subjects

All participants gave written informed consent prior to the experiments, which were approved by the local Ethics Committee and conducted in accordance with the Declaration of Helsinki.

We tested 15 patients (12 women, mean age 28.3 years, standard deviation [SD] 8.0) referred to the Epilepsy Clinic of the University Department of Neurology, Novara, Italy. All had a definite diagnosis of IGE and PPR, at the time of evaluation. Patient characteristics are summarized in Table 1. Nine of the 15 patients had a diagnosis of JME. Fourteen were seizure‐free, whereas only one patient with eyelid myoclonia with absences (EMA) reported rare absences. All but three patients were receiving standard antiepileptic treatment. A routine EEG with IPS confirmed the presence of PPR the same day prior to the experimental session. Patients were classified according to PPR distribution in posterior (type II; *n* = 4) and widespread PPR (types III and IV; *n* = 11).

**TABLE 1:** Main features of patients and photoparoxysmal response (PPR) to intermittent photic stimulation (IPS).

Patient no.	Age	Sex	Age at onset	Diagnosis	Seizure frequency	Drugs (mg)	PPR type	Range* R.	8	16	20	30	60
1P	18	F	16	IGE	Free	‐‐	W	12–20		3	3		
2P	25	F	25	IGE	Free	VPA 600	W	10–25		2–3	2		
3P	33	F	22	IGE	Free	TPM 200	W	15–25		2	2		
4P	34	M	18	JME	Free	VPA 800	P	8–20	3	3	3		
5P	21	F	11	JME	Free	LTG 250	W	10–25		3	3		
6P	25	F	18	JME	Free	VPA 1000	P	10–25		1–2–3	1–2–3		
7P	20	F	18	JME	Free	LTG 100	W	10–30		1–2	1–3	2	
8P	18	F	16	JME	Free	VPA 800	W	8–25	1–2	1–3	1–2–3		
9P	28	M	11	JME	Free	CLB 10	W	10–25		3	3		
10P	45	F	17	JME	Free	VPA 1300 LEV 1000	W	8	3				
11P	29	F	15	JME	Free	‐	W	10–25		3	3		
12P	23	F	7	IGE	Free	‐	P	16–20		3	3		
13P	40	F	12	EMA	Rare absences	PB 125	W	8–60	2	2	2	2	2
14P	34	F	8	IGE	Free	PB 150	W	14–25		2	2		
15P	32	M	12	JME	Free	LTG 150	P	8–20	3	3	3		

Abbreviations: 1, PPR occurring with open eyes; 2, on eye closure; 3, with closed eyes. W, widespread PPR. P, posterior PPR. EMA, eyelid myoclonia with absences; IGE, idiopathic generalized epilepsy; JME, juvenile myoclonic epilepsy. CLB, clobazam; LEV, levetiracetam; LTG, lamotrigine; PB, phenobarbital; TPM, topiramate; VPA, valproic acid.

* The occurrence of PPR is specified for selected IPS frequencies. The relation of PPR to the eye state is reported as well.

Twenty‐one age‐ and sex‐matched HSs served as controls (17 women, mean age 25.6 years; SD 6.3). Controls had no personal or family history of neurological disease or epilepsy and showed normal findings on general, neurological, and ophthalmological examination.

Both patients and controls refrained from neuroactive substances, including alcohol and caffeine, for 72 h prior to testing, except for ongoing antiepileptic medications in patients. Not all participants completed the full experimental protocol. Consequently, the final sample sizes were as follows: 9 patients for the 8 and 16 Hz eyes‐open condition; 13 and 15 patients for the 8 and 16 Hz eyes‐closed conditions, respectively; 20 control participants for the 8 and 16 Hz eyes‐open condition; and 21 control participants for the 8 and 16 Hz eyes‐closed condition. In addition, 9 patients and 13 controls were included in the resting condition.

### 2.2 Procedure

Participants underwent two different recordings. These included: (i) a 30‐min resting state EEG recording (*resting recordings*) with eyes closed, and (ii) intermittent photic stimulations at 8 and 16 Hz (*SSVEP recordings*). EEG was recorded with Ag/AgCl cup electrodes placed on Oz, Pz, C4, C3, Fz, according to the 10–20 International EEG System. All analyses were conducted on the occipital electrode Oz, referenced with Pz. Impedance was kept below 5 kΩ. Signal was collected with a sample rate of 10 000 Hz, amplified and bandpass filtered (1–1000 Hz) via a CED 1402 isolated amplifier (CED, Cambridge, UK), and fed to a CED Micro 1401 MK II A/D converter. Resting‐state recording analysis and results can be found in the Supplementary Material file.

#### 2.2.1 SSVEP recordings

The experiment was conducted between 2:00 and 6:30 p.m. in a quiet, dimly lit room (about 30 Lux), maintained at a standard temperature of ~22°C. Participants lay comfortably in a supine position, in a setting identical to that used for routine IPS, facing a xenon lamp (Nihon‐Kohden type 30100A), positioned 25 cm from the nasion. The lamp emitted rhythmic light pulses of 20 μs and .64 J intensity, at two temporal frequencies (8 and 16 Hz) and under two viewing conditions (eyes open and eyes closed).

Viewing conditions were presented in separate blocks in a pseudorandomized order, whereas stimulation frequencies were intermixed within each block. Each trial included 3 seconds of photic stimulation, followed by 1 second of rest period. The intertrial interval was 4 ± .7 seconds (17%), randomized to prevent habituation. Trials contaminated by oculographic, electromyography (EMG), or epileptiform artifacts were rejected online. After artifact rejection, an average of 73 ± 21 trials per participant (mean ± 1 SD) was retained for analysis.

#### 2.2.2 SSVEP: Data pre‐processing and analysis

To correct for stimulation‐related artifacts, we applied a Gaussian‐based artifact removal procedure.^19^ First, each epoch was high‐pass filtered at four times the stimulation frequency (i.e., 32 Hz for 8‐Hz stimulation and 64 Hz for 16‐Hz stimulation) to isolate the stimulation artifacts. Second, a peak detection was performed on the rectified signal. Artifacts were defined as peaks whose amplitude exceeded 5 SD above the mean of the high‐pass–filtered signal. For each detected artifact, the mean and SD of the corresponding time window (2.8 ms centered on the peak) were then estimated from the original unfiltered signal. Finally, in the original data, artifacts were replaced by random values drawn from a Gaussian distribution with the estimated mean and SD, following the method described by Hoffmann and colleagues.^19^

##### Power spectrum analysis

Spectral power was estimated for both the evoked (phase‐locked) and induced (non–phase‐locked) visual responses, focusing on the Oz channel. To minimize contamination from the initial transient response, all spectral analyses were performed on the steady‐state portion of the signal (.5–3 seconds after stimulus onset).

For the phase‐locked response, epochs were first averaged in the time domain, and a Fourier transform was then applied to the resulting signal. For the induced response, each single epoch was Fourier transformed, and the corresponding power spectra were averaged across epochs. This latter approach removes phase information, isolating power fluctuations that are stimulus‐related but not strictly time‐locked to stimulus onset. In other words, evoked responses reflect consistent, stimulus‐locked activity across trials, whereas induced responses capture modulations of ongoing oscillatory activity that vary in phase from trial to trial. Spectral energy was quantified as the average of the power spectrum over the frequency range of interest, providing a frequency‐domain estimate of signal power.

To assess the effects of group (controls vs patients), eye condition (open vs closed), and stimulation frequency (8 vs 16 Hz), we fitted a three‐way factorial linear model including the three factors and all their interactions as fixed effects. Analysis of variance (ANOVA) tests were performed using the Satterthwaite approximation for the denominator degrees of freedom.

As complementary control analyses, we normalized the single‐trial power spectra using two subject‐specific post‐stimulation reference measures computed from the eyes‐closed condition. First, for each participant at Oz, spectra were divided by the average eyes‐closed post‐stimulation (.5–1 seconds) power in the alpha range (7–13 Hz), pooling the 8 and 16 Hz stimulation frequencies. Second, spectra were divided by a broader eyes‐closed post‐stimulation scalar reference, computed by averaging post‐stimulation power across the 8 and 16 Hz stimulation frequencies and across the analyzed frequency range. In both cases, the same scalar factor was applied to the entire spectrum, preserving the frequency profile of the response while reducing the influence of subject‐level differences in global EEG amplitude.

Analyses were conducted separately for the evoked and induced amplitudes at the fundamental frequency of stimulation (f_0_) and its first harmonic (f_1_), as well as for the average decibel‐transformed power spectrum (0–35 Hz). Frequency‐wise analyses up to 35 Hz were also performed, with *p*‐values corrected for multiple comparisons using the false discovery rate (FDR; *q* = .05).^20^

We additionally performed a comparison of time‐domain signal energy between groups. Trial‐by‐trial signal energy was quantified as:

$$E=\sum_{t=1}^{T}{\left(x_{t}-\bar{x}\right)}^{2}$$

where $x_{t}$ denotes the EEG signal at time t, and T is the number of samples per trial (identical across all trials and conditions). This measure corresponds to the un‐normalized signal variance and provides a direct estimate of overall EEG signal energy in the time domain. Notably, this measure is consistent with spectral energy estimates in the frequency domain, reflecting the equivalence of time‐ and frequency‐domain representations of signal energy under Fourier transform.

##### Inter‐trial phase clustering (ITPC)

To quantify the consistency of phase across trials, we computed the inter‐trial phase clustering (ITPC) index^21^ over the Oz channel, separately for each group (control and patients) and the four experimental conditions. ITPC was calculated as:

$${\text{ITPC}}_{\tau }=\left|n^{-1}\sum_{j=1}^{n}e^{i\varphi_{\mathrm{j\tau}}}\right|$$

where n is the number of trials, $\varphi_{\mathrm{j\tau}}$ is the instantaneous phase of the signal at the time point *τ* on trial *j*.

The instantaneous phase was estimated using the Hilbert transform applied to the signal filtered around the fundamental frequency of stimulation (f_0_), using a ± 1 Hz band‐pass centered on the stimulation frequency (i.e., 7–9 Hz and 15–17 Hz for the 8 and 16 Hz stimulation conditions, respectively). ITPC was estimated as the mean ITPC between .5 and 3 seconds.

## 3 RESULTS

In the present study, we applied a clinical photo stimulation protocol at 8 and 16 Hz, under eyes‐open and eyes‐closed conditions, to elicit a steady‐state visual response in patients with IGE and PPR, as well as in healthy controls. EEG activity was recorded to investigate groups differences in both evoked and induced visual responses during photic stimulation, as well as in spontaneous resting‐state activity (see Supplementary Materials).

### 3.1 Intermittent photic stimulation: Evoked (phase‐locked) responses

We first analyzed and compared steady‐state visual evoked responses, focusing on evoked (phase‐locked) activity, across groups (controls vs patients) and experimental conditions. This analysis captures neural responses that are time‐locked to the stimulus, reflecting the consistent phase‐locked component of the visual response.

To this end, we estimated the power spectrum (PS) of the phase‐locked EEG response to photic stimulation at the Oz electrode. PS was computed for each participant and for each of the four experimental conditions (i.e., *“stimulation”* at 8 or 16 Hz, and *“eyes”* open or closed), separately. Figure 1 shows the corresponding grand‐average PS for each condition.

**FIGURE 1:**
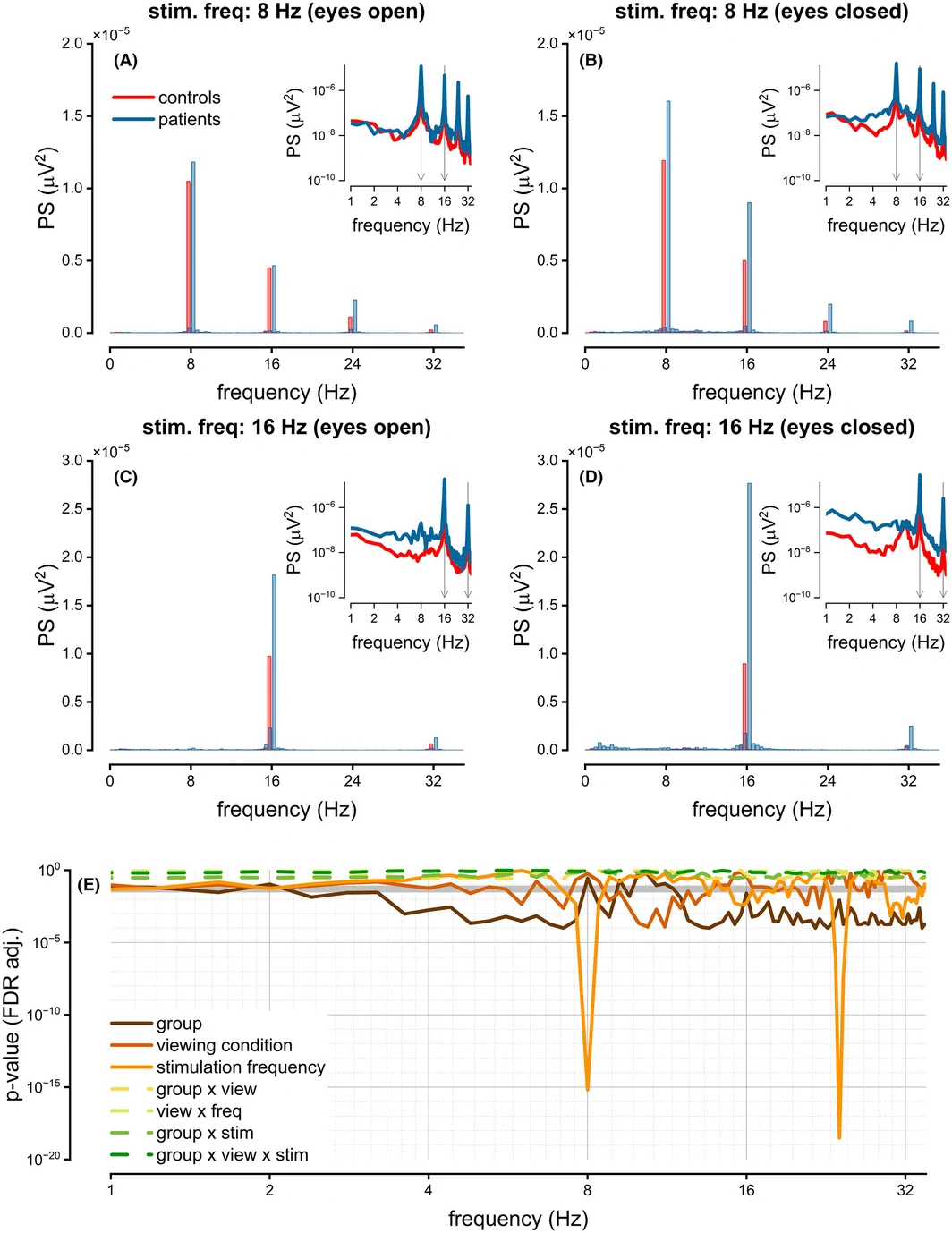
Power spectrum of the phase‐locked visual response. (A) eyes open, 8 Hz stimulation; (B) eyes closed, 8 Hz stimulation; (C) eyes open, 16 Hz stimulation; (D) eyes closed, 16 Hz stimulation. f_0_ denotes the response at the fundamental frequency of stimulation, and f_1_ denotes the first harmonic (2× stimulation frequency). In all panels, blue lines represent patients and red lines represent controls. Insets show the same data replotted in logarithmic scale to better visualize spectral differences across frequencies. (E) FDR‐adjusted *p*‐values across frequency for SSVEP measures. The horizontal gray line indicates the significance threshold (*p* < .05, FDR‐corrected). Colors denote main effects of group (dark brown), viewing condition (brown), and stimulation frequency (light brown), as well as interaction terms (dashed lines, color‐coded as indicated in the legend). PS, power spectrum; stim. freq., stimulation frequency.

Two prominent effects were observed: (i) evoked power was generally higher for patients (blue) than in controls (red), across all conditions, at both the fundamental frequency (f_0_) and its first harmonic (f_1_); (ii) power differences between groups were not restricted to the stimulation frequency, but were apparent across the entire power spectrum, as highlighted in the insets of the main panels of Figure 1.

To formally test these observations, a three‐way ANOVA (2 × 2 × 2) was conducted with the factors “*group*” (controls vs patients), “*stimulation*” (8 vs 16 Hz), and “*eyes*” (closed vs open). Analyses were performed separately for the fundamental frequency of stimulation (f_0_) and its first harmonic (f_1_), and the entire spectrum (see Methods for details).

The evoked power at the fundamental frequency (f_0_) was only marginally higher in patients compared to controls, with a main effect of group approaching significance (*F*(1,120) = 3.89, *p* = .051). No significant effects were observed for eye condition (*F*(1,120) = .61, *p* = .43) or stimulation frequency (*F*(1,120) = .50, *p* = .48), and all interaction terms were non‐significant: group × eye (*F*(1,120) = .48, *p* = .48), group × stimulation (*F*(1,120) = 1.58, *p* = .21), eye × stimulation (*F*(1,120) = .001, *p* = .96), and group × eye × stimulation (*F*(1,120) = .13, *p* = .71).

A more pronounced group effect was observed for the first evoked harmonic (f_1_), where power was significantly higher in patients than in controls (*F*(1,120) = 7.73, *p* = .006). In addition, power at f_1_ was higher for 8 vs 16 Hz stimulation (i.e., corresponding to greater power at 16 vs 32 Hz), resulting in a significant main effect of stimulation frequency (*F*(1,120) = 51.071, *p* < .001). The main effect of eye condition was non‐significant (*F*(1, 120) = 2.60, *p* = .10), and none of the interaction terms reached significance: group × eye (*F*(1,120) = 3.29, *p* = .072), group × stimulation (*F*(1,120) = .43, *p* = .50), eye × stimulation (*F*(1,120) = .84, *p* = .35), and group × eye × stimulation (*F*(1,120) = .013, *p* = .90).

The spectra shown in the insets from Figure 1 (plotted in log–log scale to facilitate comparison across the entire spectrum) suggested a generalized increase in signal energy in patients compared to controls. To test this observation, we estimated the evoked energy across the entire frequency spectrum of interest (0–35 Hz) and performed a three‐way ANOVA (2 × 2 × 2), analogous to the analyses conducted f_0_ and f_1_.

Consistent with the qualitative observation, the analysis revealed a main effect of group (*F*(1,120) = 33.17, *p* < .001), confirming higher energy in patients. Furthermore, independent of group, energy was significantly higher in the eye‐closed condition compared to eyes‐open (*F*(1,120) = 13.41, *p* < .001). The main effect of stimulation frequency was not significant (*F*(1,120) = .28, *p* = .59), and none of the interaction terms reached statistical significance: group × eye (*F*(1,120) = 2.54, *p* = .11), group × stimulation (*F*(1,120) = 1.42, *p* = .23), eye × stimulation (*F*(1,120) = .19, *p* = .66), and group × eye × stimulation (*F*(1,120) = .43, *p* = .51).

To further investigate frequency‐specific effects, we conducted the ANOVA separately for each frequency of interest (0–35 Hz) across the spectrum. Figure 1E shows the resulting *p*‐values after FDR correction, with significant frequencies indicated by points below the thick gray line (*p* < .05). Color codes represent main effects of group (dark brown), eye (brown), stimulation (light brown) and the interaction terms (dashed lines, see legend in Figure 1E).

The effect of stimulation was confined to the stimulation frequencies and their harmonics, with peaks at 8 and 16 Hz. In contrast, the group effect was widespread across frequencies, except in the alpha range (~8–12 Hz), where no significant difference between groups was observed. As expected, the eyes‐closed vs eyes‐open comparison showed a significant difference in the alpha range. No interactions between factors were significant at any frequency.

### 3.2 Intermittent photic stimulation: Induced (non–phase‐locked) responses

The presence of generalized group effect in the evoked responses, not restricted to the stimulation frequency, suggested that EEG energy differences might also be present in the induced (non–phase‐locked) activity. To test this hypothesis, we estimated the power spectrum of the non–phase‐locked EEG response at the electrode Oz, following the same procedure as for the evoked responses. Unlike evoked activity, the induced response reflects trial‐by‐trial power fluctuations while discarding phase information and coherence across trials (see Methods for details).

As for the evoked response, power spectrum was computed for each participant and each of the four tested conditions, separately. Figure 2 shows the corresponding grand‐average power spectra. Overall, induced power was generally higher for patients (blue) than healthy subjects (red), independent of the stimulation frequency. To formally assess these differences, we applied the same statistical analyses used for evoked activity.

**FIGURE 2:**
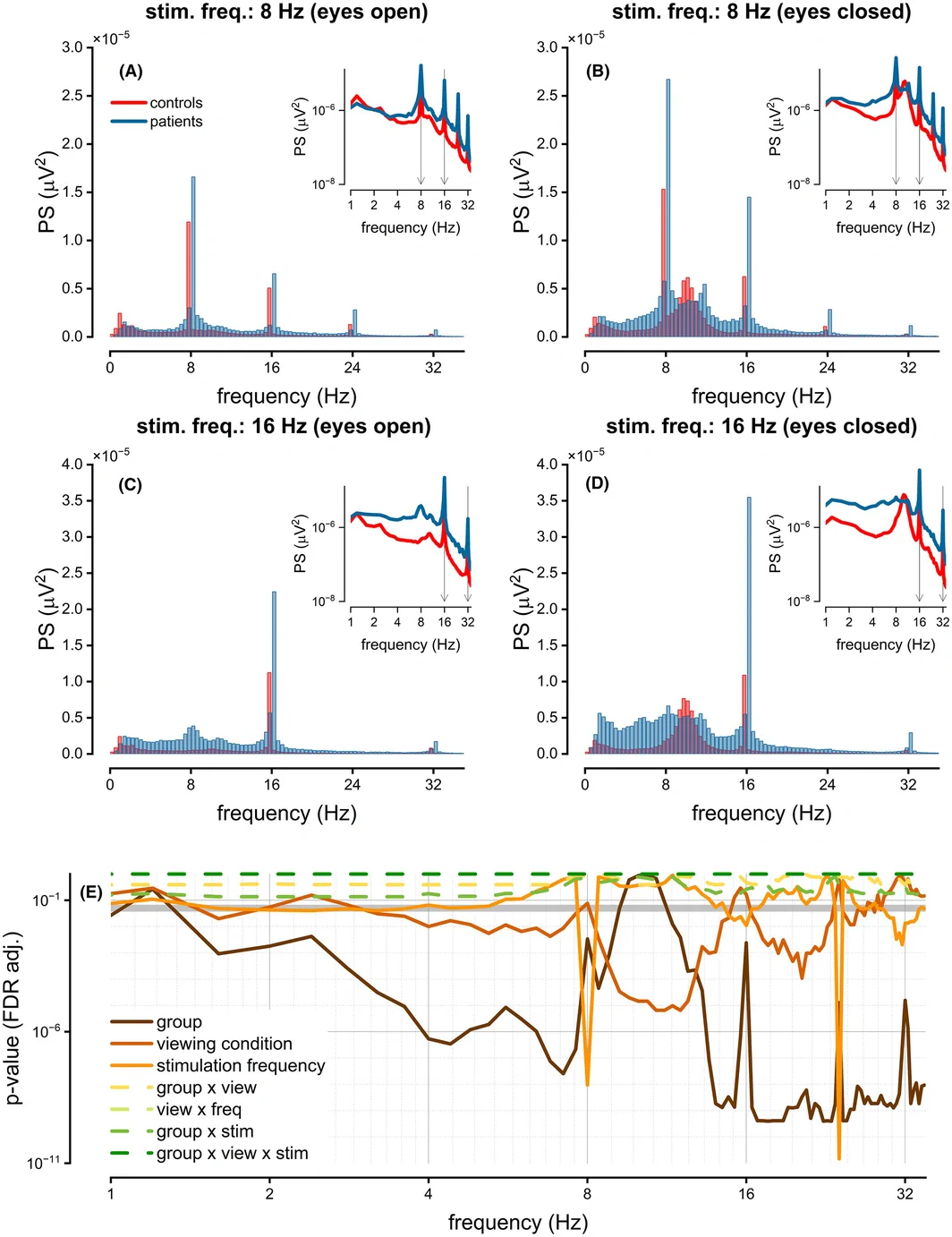
Power spectrum of the non–phase‐locked visual response. (A) eyes open, 8 Hz stimulation; (B) eyes closed, 8 Hz stimulation; (C) eyes open, 16 Hz stimulation; (D) eyes closed, 16 Hz stimulation. f_0_ denotes the response at the fundamental frequency of stimulation, and f_1_ denotes the first harmonic (2× stimulation frequency). In all panels, blue lines represent patients and red lines represent controls. Insets show the same data replotted in logarithmic scale to better visualize spectral differences across frequencies. (E) FDR‐adjusted *p*‐values across frequency for SSVEP measures. The horizontal gray line indicates the significance threshold (*p* < .05, FDR‐corrected). Colors denote main effects of group (dark brown), viewing condition (brown), and stimulation frequency (light brown), as well as interaction terms (dashed lines, color‐coded as indicated in the legend).

In contrast to the analysis of the evoked response, induced power at the fundamental frequency (f_0_) was significantly higher in patients compared to controls (*F*(1,120) = 7.83, *p* = .005). No significant effects were observed for eye condition (*F*(1,120) = 1.66, *p* = .198) or stimulation frequency (*F*(1,120) = .12, *p* = .726), and all interaction terms were non‐significant: group × eye (*F*(1,120) = .81, *p* = .368), group × stimulation (*F*(1,120) = 1.05, *p* = .307), eye × stimulation (*F*(1,120) = .03, *p* = .854), and group × eye × stimulation (*F*(1,120) = .09, *p* = .763).

Similarly to the fundamental frequency, induced power at the first harmonic (f_1_) was significantly higher in patients compared to controls (*F*(1,120) = 14.91, *p* < .001). Power at f_1_ was also higher for 8 vs 16 Hz stimulation (corresponding to greater power at 16 vs 32 Hz), resulting in a significant main effect of stimulation (*F*(1,120) = 68.45, *p* < .001). Furthermore, a significant main effect of eye condition was observed (*F*(1, 120) = 4.80, *p* = .030), indicating higher power with eyes closed compared to eyes open. A closer inspection of the condition‐specific effects indicated that induced power at f_1_ was generally higher in patients than in controls across both viewing conditions. In addition, the spectral profiles suggested a more globally elevated response in patients in both eyes‐open and eyes‐closed conditions, whereas controls showed comparatively lower and more stable power across conditions. This overall pattern was most pronounced in the eyes‐closed condition, where the group difference was largest, in line with the significant group × eye interaction (*F*(1,120) = 4.03, *p* = .046). However, this secondary interaction should be interpreted cautiously.

Post hoc analyses revealed that the group effect was most pronounced in the eyes‐closed condition, where f_1_ power in patients was significantly higher than in controls, both within the eyes‐closed and eyes‐open comparisons (*p* < .05). All other interaction terms were non‐significant: group × stimulation (*F*(1,120) = .06, *p* = .792), eye × stimulation (*F*(1,120) = 2.65, *p* = .105), and group × eye × stimulation (*F*(1,120) = .29, *p* = .588).

The spectra in the insets of Figure 2 suggested a generalized increase in induced signal energy in patients compared to healthy controls. To test this, we estimated the induced energy across the entire spectrum. Figure 2E shows the resulting FDR‐adjusted *p*‐values for each frequency. Color codes represent the main effects of group (dark brown), eye condition (brown), stimulation frequency (light brown), and interaction terms (dashed lines, see legend in Figure 2E).

The analyses revealed a residual effect of stimulation, likely reflecting the contribution of evoked activity to the induced response, which was selective for the stimulation frequencies and their harmonics, with peaks at 8 and 16 Hz. Consistent with the evoked responses, the group effect was widespread across frequencies, except in the alpha range (~8–12 Hz), where no significant difference was observed between groups. As expected, a significant difference between eyes‐closed and eyes‐open was observed in the alpha range. No interaction between factors reached significance for any frequency (dashed lines).

We additionally performed supplementary analyses to quantify trial‐by‐trial signal energy in the time domain. This analysis revealed that trial‐wise energy was significantly greater in patients than in controls (*F*(1,120) = 15.64, *p* < .001), and with the eyes closed vs open (*F*(1,120) = 9.19, *p* = .003). All other main effects and interactions were not statistically significant (*p*'s >.063). This result supports the interpretation that the EEG signal in patients is characterized by generally higher variance than in healthy controls.

To assess whether the group difference could be explained by subject‐level differences in global EEG amplitude, we performed two post‐stimulation normalization analyses using eyes‐closed post‐stimulation activity as reference. Alpha‐band normalization yielded a pattern qualitatively similar to the non‐normalized spectra, with patients showing higher normalized power across a broad portion of the spectrum, particularly outside the alpha range (Figure S1). A broader eyes‐closed post‐stimulation scalar normalization produced a similar result: patients showed higher normalized power across much of the spectrum, especially from ~4 Hz upward, with reduced or absent differences in the alpha range (Figure S2). These control analyses suggest that the group difference is not solely explained by global amplitude scaling, although they do not fully separate stimulation‐driven responses from ongoing spectral differences.

### 3.3 Intermittent photic stimulation: Inter‐trial phase clustering (ITPC)

A direct comparison of evoked and induced responses revealed that the differences between patients and controls were not limited to the stimulation frequencies or their harmonics but were instead generalized across the entire spectrum. Partial‐eta‐squared values (η_p_^2^) were estimated from the ANOVA and correspond to the average effect size across all tested frequencies.

Of note, the group effect was more pronounced for the induced response than for the evoked response, as reflected by a large effect size for the induced response (η_p_^2^ = .206 ± .01, mean and standard error of the mean [SEM]) compared to a moderate effect size for the evoked response (η_p_^2^ = .09 ± .004).

The stronger effect observed in the induced response suggests that patient's exhibit higher response amplitude, but lower phase coherence compared to controls. Conceptually, two groups responding to 8 Hz stimulation—one with high‐energy, low‐phase‐coherence signals and the other with low‐energy, high‐phase‐coherence signals—can show nearly identical phase‐locked averages despite different trial‐to‐trial variability. The evoked response reflects only the phase‐consistent component, whereas the induced response, averaging signal amplitude regardless of phase, captures total energy, including phase‐inconsistent contributions, and is therefore higher in the high‐energy, low‐phase‐coherence group. Applied to our data, this suggests that the stronger group effect in the induced response reflects a general reduction in phase coherence, alongside overall stronger visual responses in patients.

We tested this hypothesis by estimating the ITPC at the fundamental frequency of stimulation (f_0_), as a measure of phase‐variability across trials. ITPC values range from 0 (low phase coherence) to 1 (high phase coherence), and Figure 3 shows the ITPC for all conditions.

**FIGURE 3:**
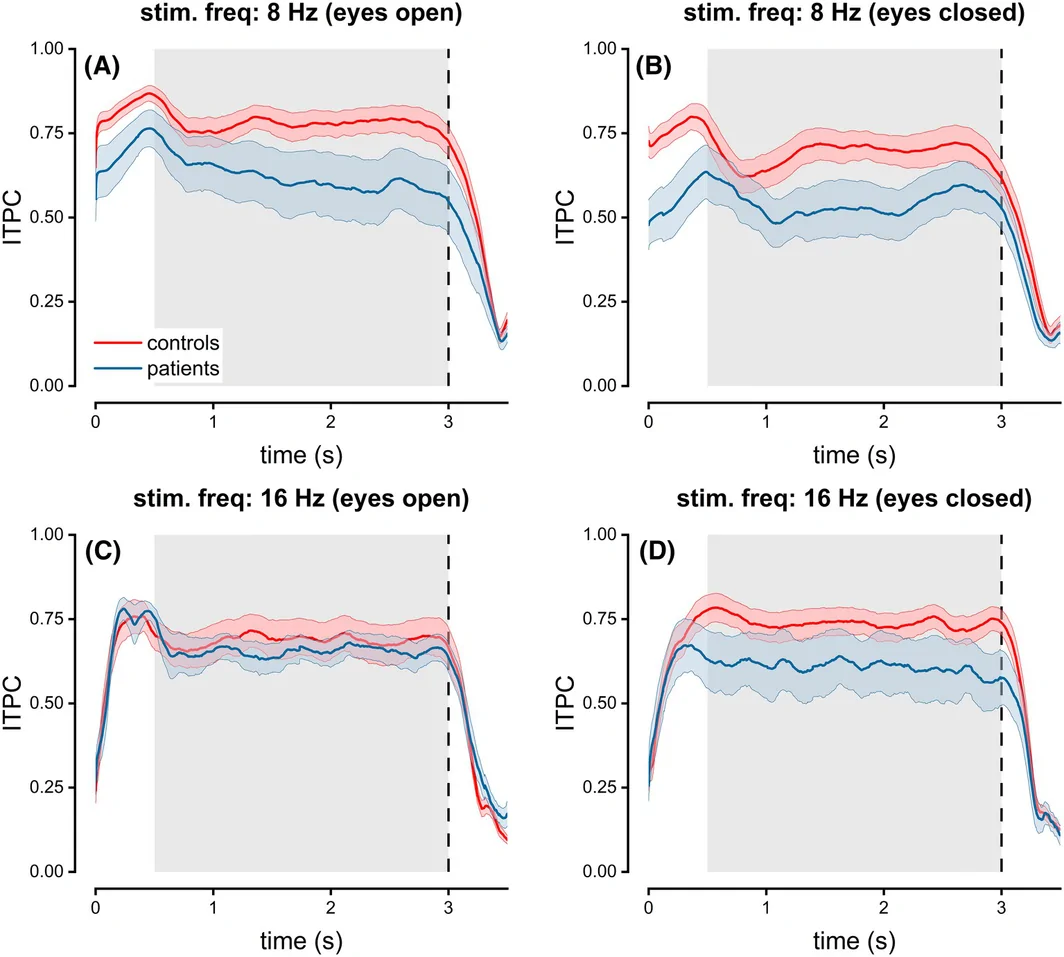
Inter‐trial phase clustering (ITPC) at the fundamental frequency of stimulation (f_0_). ITPC time courses are shown for each condition: (A) eyes open, 8 Hz stimulation; (B) eyes closed, 8 Hz stimulation; (C) eyes open, 16 Hz stimulation; and (D) eyes closed, 16 Hz stimulation. Blue lines indicate patients, and red lines indicate controls. Shaded areas represent ± 1 standard error of the mean (SEM). ITPC values range from 0 (low phase coherence) to 1 (high phase coherence). Across all conditions, patients exhibited lower ITPC than controls, reflecting reduced phase coherence and thus greater phase variability across trials.

A three‐way (2 × 2 × 2) ANOVA examining the effects of group (patients vs controls), stimulation (8 vs 16 Hz), and eyes (open vs closed) revealed a significant main effect of group (*F*(1,120) = 9.21, *p* = .002), with no significant main effects of eye (*F*(1,120) = 1.21, *p* = .27) or stimulation frequency (*F*(1,120) = .14, *p* = .70), or any significant interaction: group × eye (*F*(1,120) = .53, *p* = .46), group × stimulation (F(1,120) = .87, *p* = .35), eye × stimulation (*F*(1,120) =1.06, *p* = .30), and group × eye × stimulation (*F*(1,120) = .30, *p* = .58). These results indicate that patients exhibited higher phase variability (lower ITPC) than controls, complementing the differences observed in evoked and induced response.

## 4 DISCUSSION

In this study, we investigated visual cortical excitability in patients with IGE and photosensitivity by means of SSVEPs at two stimulation frequencies, as well as resting‐state EEG activity (for the resting‐state analysis, see Figure S3). Compared to healthy subjects, patients exhibited increased EEG power both at rest and during visual stimulation, accompanied by reduced ITPC. This pattern indicates that patients have stronger but less temporally synchronized neural responses. This dissociation between power and phase coherence was evident when comparing phase‐locked (evoked) and non–phase‐locked (induced) responses and extended to the spontaneous activity recorded in resting state. Together, these findings indicate that photosensitive epilepsy is characterized not only by increased cortical excitability but also by altered temporal coordination of visual cortical responses. Because all analyses were performed at electrode Oz, the present data do not allow direct conclusions about large‐scale network dynamics. Nevertheless, the convergence between stimulation‐related and resting‐state measures suggests a broader alteration of cortical excitability and temporal stability that should be further tested with spatially distributed recordings.

Phase‐locked responses showed increased power at stimulation harmonics, whereas the concurrent reduction in ITPC indicates that cortical activity, although amplified, is less temporally precise and less effectively entrained by rhythmic input. In parallel, induced responses revealed a further amplification of non–phase‐locked activity, indicating that oscillatory responses extend beyond strictly phase‐locked entrainment and reflect enhanced ongoing network reverberations.

Resting‐state findings further support this interpretation, with increased broadband power and altered alpha dynamics consistent with a state of elevated baseline excitability and reduced temporal stability, independent of external stimulation. Of note, similar patterns of variability in background EEG activity have been reported in genetically influenced forms of idiopathic generalized epilepsy, although their direct mapping onto individual clinical phenotypes remains challenging.^22^

Together, these results support a pathophysiological framework in which increased cortical excitability is coupled with impaired temporal precision, leading to a system that is more prone to aberrant synchronization when driven by rhythmic visual input. These findings are consistent with alterations in visual cortical dynamics that may reflect broader network‐level involvement, although spatially distributed recordings would be required to directly test network interactions.

Our findings align with prior studies reporting increased amplitude of VEPs in patients with photosensitivity compared to controls. Strigaro et al.,^10^ Lee et al.^23^ and Brinciotti et al.^24^ all reported enhanced VEP responses in patients, supporting the notion of visual cortical hyperreactivity in photosensitivity. More recently, similar findings have been obtained using SSVEP paradigms^17^, ^25^ further confirming that steady‐state stimulation elicits abnormally amplified cortical responses in photosensitive individuals.

Our findings are broadly in line with those reported by Timar et al.^16^; however, the limited number of trials in their study—based on single continuous stimulation trains of 7 seconds per frequency—may have reduced sensitivity to more subtle differences, particularly between patients with milder photosensitivity and healthy controls. In addition, this design does not allow a clear dissociation between increased response magnitude and altered synchronization dynamics. In contrast, our trial‐based approach, using multiple brief repeated stimulations, was specifically aimed at enhancing sensitivity to the hyperexcitability endophenotype and at disentangling amplitude‐related and phase‐related components of the response. On the other hand, the study by Timar et al. includes a network‐level analysis that is not captured by our single‐channel approach, representing a limitation of the present work and an important direction for future investigations.

The relationship between amplitude enhancement and phase synchronization remains complex. Although amplitude‐based metrics have been studied extensively, investigations into phase dynamics are comparatively limited. Kalitzin et al.^26^ suggested that cortical excitability could be evaluated by assessing phase clustering across trials. They reported increased phase synchronization at higher harmonic frequencies during PPRs. Similarly, Bauer et al.^25^ observed higher phase coherence in drug‐free patients with JME compared to healthy controls. However, Vranic‐Peters et al.^17^ highlighted considerable individual variability in phase coherence, which depended on the frequency band analyzed, the electrode pairs considered, and temporal proximity to paroxysmal events.

Of interest, our results suggest a distinct pattern: even in the absence of clinical PPRs or seizures, photosensitive patients show increased signal power but reduced phase coherence at stimulation frequencies. This pattern may reflect an early stage of network instability, characterized by elevated excitability but impaired temporal coordination, potentially predisposing the system to paroxysmal discharges under more intense or prolonged stimulation.

Finally, Bhagat et al.^27^ offered an alternative perspective by assessing the entropy of brain responses to visual stimuli, reporting increased unpredictability in patients with epilepsy. Although their study was limited in sample size, these findings support the hypothesis that disorganized cortical dynamics, rather than mere hypersynchrony, may underlie pathological sensory processing.

Our findings indicate that photosensitive epilepsy is characterized not merely by an exaggerated cortical response to visual stimuli, but by a fundamental alteration in the brain's ability to coordinate its activity over time. This impaired temporal synchrony may lower the threshold for pathological network synchronization under rhythmic sensory drive, thereby facilitating the emergence of PPRs.

By examining both evoked and induced responses, alongside resting‐state activity, we show that cortical instability in photosensitive epilepsy is not confined to periods of stimulation but rather reflects a more pervasive feature of network dynamics. This broader perspective may have implications for identifying functional biomarkers of network vulnerability in epilepsy, independent of spontaneous paroxysms.

Several limitations should be acknowledged. First, although SSVEPs provide a rich measure of ongoing network activity, they may not capture all dimensions of cortical excitability. Prior work by Porciatti et al.^14^ demonstrated impaired inhibitory control in photosensitive patients using contrast‐dependent visual stimulation paradigms, highlighting complementary aspects of cortical dysfunction not directly assessed by our approach.

Second, the present study focused specifically on patients with IGE and photosensitivity. Although these results provide insights into the cortical dynamics underlying photosensitive epilepsy, caution is needed in generalizing them to other epilepsy syndromes. Different epileptic conditions may involve distinct mechanisms of excitability and network instability, and it remains to be determined whether SSVEP‐derived measures exhibit similar patterns across diverse clinical populations. Future studies should therefore aim to replicate and expand these findings in a broader range of epilepsies, including focal epilepsies and genetic generalized epilepsy without photosensitivity.

Third, most of our patients were taking anti‐seizure medications at the time of testing. Although none experienced seizures during the EEG sessions, and the stimulation parameters used were within safe limits, we cannot exclude the possibility that pharmacological effects influenced cortical excitability, synchronization dynamics, or resting‐state oscillatory properties. Future investigations involving drug‐naive patients, or longitudinal designs assessing the impact of medication, would help disentangle the contributions of intrinsic network properties vs treatment‐related modulation.

An important limitation is that group differences during photic stimulation cannot be interpreted as fully independent of group differences in ongoing EEG activity. Although recordings were acquired using identical hardware, acquisition settings, and electrode preparation procedures, these controls do not exclude genuine group‐related differences in baseline spectral activity. Indeed, resting‐state analyses showed higher broadband power and greater alpha‐peak variability in patients. For this reason, we interpret the absolute and normalized spectra as complementary measures. Absolute power captures the overall magnitude of EEG activity during stimulation, whereas the normalization analyses test whether group differences persist after scaling spectra by each participant's own post‐stimulation activity. The persistence of higher normalized power in patients suggests that the effect is not solely due to global amplitude scaling. However, these analyses do not fully disentangle stimulation‐driven responses from baseline spectral differences. The findings should therefore be interpreted as evidence of altered cortical excitability and synchronization dynamics in the context of broader differences in the EEG spectral profile.

Finally, although our results support a simplified theoretical model linking hyperexcitability with impaired synchronization, it is evident that epileptogenic network dynamics are highly complex. Multimodal approaches that combine visual evoked potential with complementary stimulation and recording techniques will be essential for fully elucidating how cortical circuits transition from physiological instability to seizure generation.

## AUTHOR CONTRIBUTIONS

R.C. and G.S. conceived and designed the study and collected the data. A.B., C.S., and F.T. analyzed the data. All authors contributed to writing and critically revising the manuscript and approved the final version.

## FUNDING INFORMATION

This study received no specific funding.

## CONFLICT OF INTEREST STATEMENT

The authors declare no conflicts of interest related to this study.

## ETHICAL PUBLICATION STATEMENT

We confirm that we have read the Journal's position on issues involved in ethical publication and affirm that this report is consistent with those guidelines.

## Supporting information


Figure S1.
Figure S2.
Figure S3.

## Data Availability

The data that support the findings of this study are available from the corresponding author upon reasonable request.
