# Supplementary material for: Altered cortical synchronization in photosensitive idiopathic generalized epilepsy
Source: Epilepsia. 2026 Aug 13;67(11):5959–72. doi: 10.1002/epi.70425 (PMC13768895; doi:10.1002/epi.70425)
Supplement: Supplementary file 1 [file EPI-67-5959-s001.docx]

## Supplementary Methods

### Resting state: data pre-processing and analysis

#### Power spectrum analyses

Spectral power was estimated, over the entire recording, using the Welch method (3 s window length, 50% overlap, Hann window, with 15 s zero-padding). The spectrum was computed as the average of O1 and O2 electrodes.

Consistent with the SSVEP analysis, the average decibel-transformed power spectrum (averaging the power spectrum up to 35 Hz) was compared between the two groups using a two-sample t-test assuming unequal variance. Frequency-wise analyses were also performed across the 0-35 Hz range, with resulting p-values corrected for multiple comparisons using the false discovery rate (FDR; q = 0.05).

#### Individual alpha peak frequency (IAPF) analyses

Spectral power was estimated, over the entire recording, using the Welch method (3 s window length, 50% overlap, Hann window, with 15 s zero-padding). The spectrum was computed as the average of O1 and O2 electrodes.

Consistent with the SSVEP analysis, the average decibel-transformed power spectrum (averaging the power spectrum up to 35 Hz) was compared between the two groups using a two-sample t-test assuming unequal variance. Frequency-wise analyses were also performed across the 0-35 Hz range, with resulting p-values corrected for multiple comparisons using the false discovery rate (FDR; q = 0.05).

#### Normalized single-trial spectral responses

As complementary control analyses, we normalized the single-trial power spectra using two subject-specific post-stimulation reference measures computed from the eyes-closed condition. First, we performed an alpha-band reference normalization. For each participant at Oz, spectra were divided by the average eyes-closed post-stimulation power in the alpha range (7–13 Hz), computed over the 0.5–1 s post-stimulation interval and pooled across the 8 and 16 Hz stimulation frequencies.

Second, we performed a broader post-stimulation scalar normalization. For each participant at Oz, spectra were divided by a single eyes-closed post-stimulation reference value obtained by averaging post-stimulation power across the 8 and 16 Hz stimulation frequencies and across the analyzed frequency range. In both analyses, the same scalar factor was applied to the entire spectrum, preserving the frequency profile of the response while reducing the influence of subject-level differences in global EEG amplitude.

## Supplementary Results

### **Normalized single-trial spectral responses**

Alpha-band reference normalization yielded a pattern qualitatively similar to the unnormalized spectra. Patients showed higher normalized power across a broad portion of the spectrum, particularly outside the alpha range (Figure S1). This analysis suggests that the group difference is not solely explained by global amplitude scaling, although the alpha reference should be interpreted cautiously because alpha activity is state-dependent and showed greater variability in patients.

***Figure S1. Power spectrum of the alpha-band-normalized single-trial visual response.*** ***A****, eyes open, 8 Hz stimulation;* ***B****, eyes closed, 8 Hz stimulation;* ***C****, eyes open, 16 Hz stimulation;* ***D****, eyes closed, 16 Hz stimulation. f0 denotes the response at the fundamental frequency of stimulation, and f1 denotes the first harmonic (2× stimulation frequency). In all panels, blue lines represent patients and red lines represent controls. Insets show the same data replotted in logarithmic scale.* ***E****, FDR-adjusted p-values across frequency. The horizontal gray line indicates the significance threshold (p < 0.05, FDR-corrected). Colors denote main effects of group, viewing condition, and stimulation frequency, as well as interaction terms.*

The broader post-stimulation scalar normalization produced a similar result. Patients continued to show higher normalized power across much of the spectrum, especially from approximately 4 Hz upward, with reduced or absent differences in the alpha range (Figure S2). Because this normalization used a single scalar reference for each participant, it preserved spectral-shape differences while reducing subject-level differences in overall post-stimulation amplitude.

***Figure S2. Power spectrum of the post-stimulation scalar-normalized single-trial visual response. A****, eyes open, 8 Hz stimulation;* ***B****, eyes closed, 8 Hz stimulation;* ***C****, eyes open, 16 Hz stimulation;* ***D****, eyes closed, 16 Hz stimulation. f_0_ denotes the response at the fundamental frequency of stimulation, and f_1_ denotes the first harmonic (2× stimulation frequency). In all panels, blue lines represent patients and red lines represent controls. Insets show the same data replotted in logarithmic scale.* ***E****, FDR-adjusted p-values across frequency. The horizontal gray line indicates the significance threshold (p < 0.05, FDR-corrected). Colors denote main effects of group, viewing condition, and stimulation frequency, as well as interaction terms.*

Together, these normalization analyses support the interpretation that the group difference is not solely due to global EEG amplitude scaling. However, because both procedures use post-stimulation activity as a reference, they should be interpreted as complementary control analyses rather than as a complete separation of stimulation-driven responses from ongoing spectral differences.

### Resting state: spontaneous activity and IAPF

The results presented so far suggest that the spectral differences between patients and controls were not strictly related to visual stimulation but may instead reflect differences in ongoing (spontaneous) cortical activity. To account for this hypothesis, we analyzed the resting-state power spectrum in both groups. As shown in Supplementary Figure S3, the spontaneous EEG activity was generally higher in patients than in controls (two-sample t-test, assuming unequal variance: t(19.8) = -2.55, p = 0.019). Consistent with the evoked and induced response results, the group difference was significant across most of the frequency spectrum, except in the alpha range (and its harmonics), where no significant differences were observed between groups

***Figure S3 – Resting-state power spectrum and alpha peak variability. A****, Average power spectrum of resting-state EEG activity (eyes closed) for patients (blue) and healthy controls (red). Shaded areas represent ±1 standard error of the mean (s.e.m.). Thick black lines at the bottom indicate frequencies showing significant group differences (p < 0.05, FDR-corrected). Patients exhibited higher overall EEG power compared to controls, except in the alpha range (~7–13 Hz), where no significant differences were found.* ***B****, Within-subject variability of the Individual Alpha Peak Frequency (IAPF), quantified as the standard deviation across temporal windows. Patients showed significantly greater IAPF variability than controls, indicating less stable alpha oscillations.*

Building on the findings and hypotheses supported by the ITPC analyses – specifically, that patients’ EEG activity is characterized by overall increased power, but greater phase variability compared to controls – we hypothesized that the absence of a clear group effect in the alpha range might be due to increased variability in alpha activity among patients. To test this, we estimated the Individual Alpha Peak Frequency (IAPF) and examined its within-subject variability, quantified as the standard deviation across temporal windows. We found that this variability was significantly higher in patients than controls (two-sample t-test with unequal variance: t(17.4) = -3.29, p = 0.004). In other words, the alpha peak frequency was markedly more unstable in patients, which could explain the relative attenuation of the alpha response observed in this group. Moreover, between-subject comparisons revealed that the variability of IAPF across patients was also significantly greater than that across controls (two-sample F-test, two-tailed: F(8,12) = 0.18, p = 0.022).
